# Short Rest Between Flights Is Associated With Increased Serum Stress Hormone Levels in Airline Pilots: A Cross-Sectional Study

**DOI:** 10.7759/cureus.69505

**Published:** 2024-09-16

**Authors:** Piercarlo Minoretti, Davide D’Acquino, Konstantinos Lavdas, Giovanni Fortuna

**Affiliations:** 1 Occupational Health, Studio Minoretti, Oggiono, ITA

**Keywords:** airline pilots, biomarkers, cortisol, dehydroepiandrosterone-sulfate, occupational stress, rest periods, stress hormones

## Abstract

Background: Work-related stress is a critical area of research in civil aviation, given the potential for severe consequences when airline pilots (APs) are overwhelmed or unable to perform optimally. While pilots are traditionally considered to be exposed to various stressors, the impact of specific occupational characteristics on stress in the aviation industry remains inadequately understood. Considering that biomarkers are increasingly being utilized as objective measures of stress in human research, this cross-sectional study investigated the association between occupational variables and serum levels of cortisol and dehydroepiandrosterone sulfate (DHEAS) as stress biomarkers in commercial APs.

Methods: A total of 120 male APs completed a survey assessing the following work-related characteristics: position (captain vs. first officer), years of experience, total flight hours, flight hours in the preceding year, inter-flight rest duration, and flight route length. Serum cortisol and DHEAS concentrations were determined from venous blood samples obtained between 08:00 and 09:00 a.m., following a minimum eight-hour fasting period. The biomarker data were analyzed in relation to the aforementioned occupational characteristics.

Results: The mean serum cortisol and DHEAS concentrations in the entire cohort were 8.5±2.1 µg/dL and 214.6±96.4 µg/dL, respectively. There were no significant differences in the levels of the two stress hormones in relation to position, years of experience, total flight hours, flight hours in the preceding year, or flight route length. However, an inter-flight rest period of less than one hour was significantly associated with elevated serum cortisol (P<0.01) and reduced DHEAS levels (P<0.001) compared to longer rest durations. Notably, a stepwise decrease in DHEAS concentrations was observed for rest periods of <1 hour, 1-4 hours, 4-24 hours, and >24 hours. After adjustment for potential confounders in multivariable analyses, a rest period of <1 hour remained independently associated with both serum cortisol (odds ratio [OR]=1.09, 95% confidence interval [CI]=1.04-1.13, P<0.01) and DHEAS (OR=0.94, 95% CI=0.92-0.97, P<0.001).

Conclusions: Serum stress hormones in APs are associated with short inter-flight rest periods. Optimization of rest durations may contribute to improved pilot well-being and performance. Further research is warranted to determine ideal rest period lengths and develop interventions to mitigate the potential adverse effects of abbreviated rest periods between flights.

## Introduction

The aviation industry presents a unique and challenging work environment, characterized by irregular schedules, complex operational demands, and high-stakes decision-making processes that can engender significant occupational stress [1]. In recent years, there has been a burgeoning interest in work-related stress among airline pilots (APs), given its potential ramifications for both flight safety and pilot well-being [2-5]. Regarding the former, stress-induced impairments in cognitive function, decision-making capabilities, and situational awareness can precipitate errors that compromise flight safety [3-5]. Concerning the latter, chronic stress exposure has been associated with deleterious long-term health consequences for pilots, including fatigue, sleep disturbances, and depressive symptomatology [6]. These health concerns not only affect individual pilots but also have broader implications for the aviation industry, including increased absenteeism [6] and diminished job satisfaction [5].

Growing evidence has underscored the utility of biomarkers as objective measures of psychophysiological stress responses [7]. These molecules offer a more precise and quantifiable assessment of stress levels compared to subjective self-reports, which may be influenced by individual perceptions and reporting biases [7]. Cortisol and dehydroepiandrosterone sulfate (DHEAS) are two biomarkers of particular interest in stress research [8,9]. Cortisol is a crucial endogenous glucocorticoid that plays a central role in the body's stress response system [10]. It is produced by the adrenal glands and regulates various physiological processes, including metabolism, immunity, cognitive functions, and circadian rhythm [10,11]. DHEAS, another adrenal hormone, has gained increasing attention in stress research due to its potential as a biomarker of chronic stress and resilience [9]. DHEAS is the sulfated form of dehydroepiandrosterone, which is a precursor to both androgens and estrogens [12]. This hormone plays a crucial role in neuroprotection, immune function, and maintaining overall physiological balance [13]. Unlike cortisol, DHEAS levels typically decrease in response to prolonged stress [14,15]. Collectively, these two biomarkers offer valuable insights into the human stress response, providing a comprehensive assessment of psychophysiological stress states [8].

While prior research has explored the subjective effects of work-related stress on APs [2-4,16], there is a paucity of studies examining the relationship between specific occupational factors and circulating stress hormones. To address this gap, we conducted a cross-sectional study to investigate potential associations between key work-related variables and serum concentrations of cortisol and DHEAS in a sample of commercial pilots. We hypothesized that specific occupational characteristics, such as flight hours, work schedules, and job seniority, would be significantly related to alterations in serum stress hormones.

## Materials and methods

Study participants

The study sample comprised 120 male APs of Caucasian descent, all in satisfactory physical health, who voluntarily participated in the research. Consistent with previous studies [17-19], recruitment was restricted to male subjects due to the limited number of female pilots. None of the participants had psychiatric, neurological, endocrine, infectious, autoimmune, or malignant conditions. Additionally, smokers and individuals who had used steroids in the past three months were excluded. Recruitment took place during routine occupational health visits at outpatient clinics (Studio Minoretti, Oggiono, Italy), where an experienced occupational health physician invited pilots to participate. The research protocol was approved by the local ethics committee (Studio Minoretti, reference number: 2021/07SH), and written informed consent was obtained from all participants.

Data collection

Anthropometric measurements were conducted using standardized protocols. Participants' stature was assessed to the nearest 0.1 cm using a wall-mounted stadiometer, while body mass was determined to the nearest 0.1 kg with a calibrated digital scale. Subjects were instructed to remove footwear and heavy outer garments before measurements. The body mass index (BMI) was calculated by dividing the weight in kilograms by the square of the height in meters. After a minimum rest period of 5-10 minutes in a seated position, blood pressure was measured using an automated digital sphygmomanometer (Lotus Global Co., Ltd., London, UK). Educational background was quantified as the total number of years of formal schooling completed by each participant.

Work-related variables

A comprehensive survey was conducted to gather data on several key factors related to the pilots’ professional experience and working conditions. Participants were asked to provide information about their current position, categorized as either captain or first officer. Pilots' years of experience were divided into three categories: 0−10 years, 11−15 years, and more than 15 years. To assess flight experience, participants reported their total flying hours, categorized into four groups: less than 3,000 hours, 3,000−5,000 hours, 5,000−10,000 hours, and more than 10,000 hours. Pilots were also asked about their flying hours in the past year, with responses grouped into three categories: less than 500 hours, 500−700 hours, and more than 700 hours. We further explored aspects of work scheduling and flight patterns. Accordingly, participants provided information on their typical rest time between flights, which was categorized into four intervals: <1 hour, 1−4 hours, 4−24 hours, and more than 24 hours. Lastly, the survey collected data on the typical duration of flight routes, with responses grouped into three categories: less than six hours, 6−12 hours, and more than 12 hours.

Laboratory methods

Venous blood samples were collected from an antecubital vein between 08:00 and 09:00 a.m. to minimize diurnal variations, following a minimum eight-hour fasting period. The serum was prepared via centrifugation at 3,500 rpm for 15 min, and cortisol and DHEAS levels were measured using a Cobas 6000 Analyzer (Roche Diagnostics, Basel, Switzerland). All measurements were performed in duplicate by personnel unaware of participants' characteristics to minimize bias.

Statistical analysis 

Descriptive statistics, including means, standard deviations, frequencies, and percentages, were employed to summarize the dataset comprehensively. To compare serum stress hormone concentrations between two groups, we utilized the Student's *t*-test. For comparisons involving three or more groups, we applied one-way analysis of variance (ANOVA). Upon identifying statistically significant differences between groups through ANOVA, we conducted *post hoc* multiple comparisons using Tukey's tests. Additionally, we performed a *post hoc* analysis for linear trends in ANOVA to test the null hypothesis that no linear trend exists between the means and the group order. Pearson's correlation coefficients were calculated to evaluate the univariate associations between serum cortisol and DHEAS levels and the participants' characteristics. The independent relationship between an inter-flight rest period of less than one hour (exposure) and serum stress hormone levels was examined using multivariable logistic regression, adjusting for age, BMI, years of education, and blood pressure values. Results are presented as odds ratios (ORs) accompanied by their respective 95% confidence intervals (CIs). Statistical analyses were performed using IBM SPSS Statistics for Windows, Version 20.0 (Released 2011; IBM Corp., Armonk, NY, USA). Results were considered statistically significant at a two-sided alpha level of 0.05. Given the exploratory nature of this study, no adjustments were made for multiple comparisons.

## Results

General characteristics of the study participants

The study cohort had an average age of 42 years, with a standard deviation of six years. The pilots' mean BMI was 24 kg/m², with a standard deviation of 4 kg/m². On average, participants had completed 17 years of education, with a standard deviation of five years. The mean systolic blood pressure was 122 mmHg, with a standard deviation of 27 mmHg, while the mean diastolic blood pressure was 75 mmHg, with a standard deviation of 10 mmHg.

Serum stress hormones in relation to work-related variables

The mean serum cortisol and DHEAS concentrations in the entire cohort were 8.5±2.1 µg/dL and 214.6±96.4 µg/dL, respectively. Serum cortisol and DHEAS levels showed a negative correlation in the entire study sample (Pearson's correlation coefficient r = -0.29, P<0.05); however, none of the stress hormones showed significant associations with age, BMI, years of education, and blood pressure values. The mean cortisol and DHEAS levels in relation to pilots’ work-related variables are presented in Table 1.

**Table 1:** Serum stress hormone levels in relation to pilots' work-related variables. Data presented as counts (percentages), means, and SDs, as indicated. ^a^P value for comparison of mean cortisol levels between groups. ^b^P value for comparison of mean DHEAS levels between groups. *P values were calculated using one-way ANOVA. In all analyses, P<0.05 was considered statistically significant; NS: not significant (P≥0.05). ANOVA: analysis of variance; SD: standard deviation; DHEAS, dehydroepiandrosterone sulfate.

Occupational information	Category	Count (%)	Mean cortisol (µg/dL)	SD cortisol (µg/dL)	Mean DHEAS (µg/dL)	SD DHEAS (µg/dL)	P value^a^	P value^b^
Position	Captain	54 (45%)	8.3	2.0	217.3	95.0	NS*	NS*
	First officer	66 (55%)	8.7	2.2	212.2	97.0		
Years of experience	0–10	69 (57.5%)	8.5	2.1	215.8	96.0	NS*	NS*
	11–15	18 (15%)	8.4	2.0	214.5	95.5		
	>15	33 (27.5%)	8.6	2.1	218.7	97.5		
Flying hours (total)	<3,000	68 (56.67%)	8.5	2.1	214.9	96.2	NS*	NS*
	3,000–5,000	21 (17.5%)	8.4	2.0	215.2	96.5		
	5,000–10,000	22 (18.33%)	8.7	2.2	217.8	97.8		
	>10,000	9 (7.5%)	8.5	2.1	212.5	94.0		
Flying hours (past year)	<500	35 (29.17%)	8.6	2.1	216.3	96.8	NS*	NS*
	500–700	48 (40%)	8.7	2.2	214.9	96.4		
	>700	37 (30.83%)	8.4	2.0	213.9	95.8		
Rest time between flights	<1 hour	60 (50%)	8.9	2.3	202.2	90.0	<0.001*	<0.001*
	1–4 hours	26 (21.67%)	8.2	1.9	216.5	96.7		
	4–24 hours	22 (18.33%)	8.1	1.8	221.7	99.0		
	>24 hours	12 (10%)	8.3	2.0	228.3	102.0		
Flight routes duration	<6 hours	97 (80.83%)	8.4	2.1	212.1	95.0	NS*	NS*
	6–12 hours	13 (10.83%)	8.4	2.0	216.3	96.5		
	>12 hours	10 (8.34%)	8.7	2.2	214.8	96.2		

**Figure 1:**
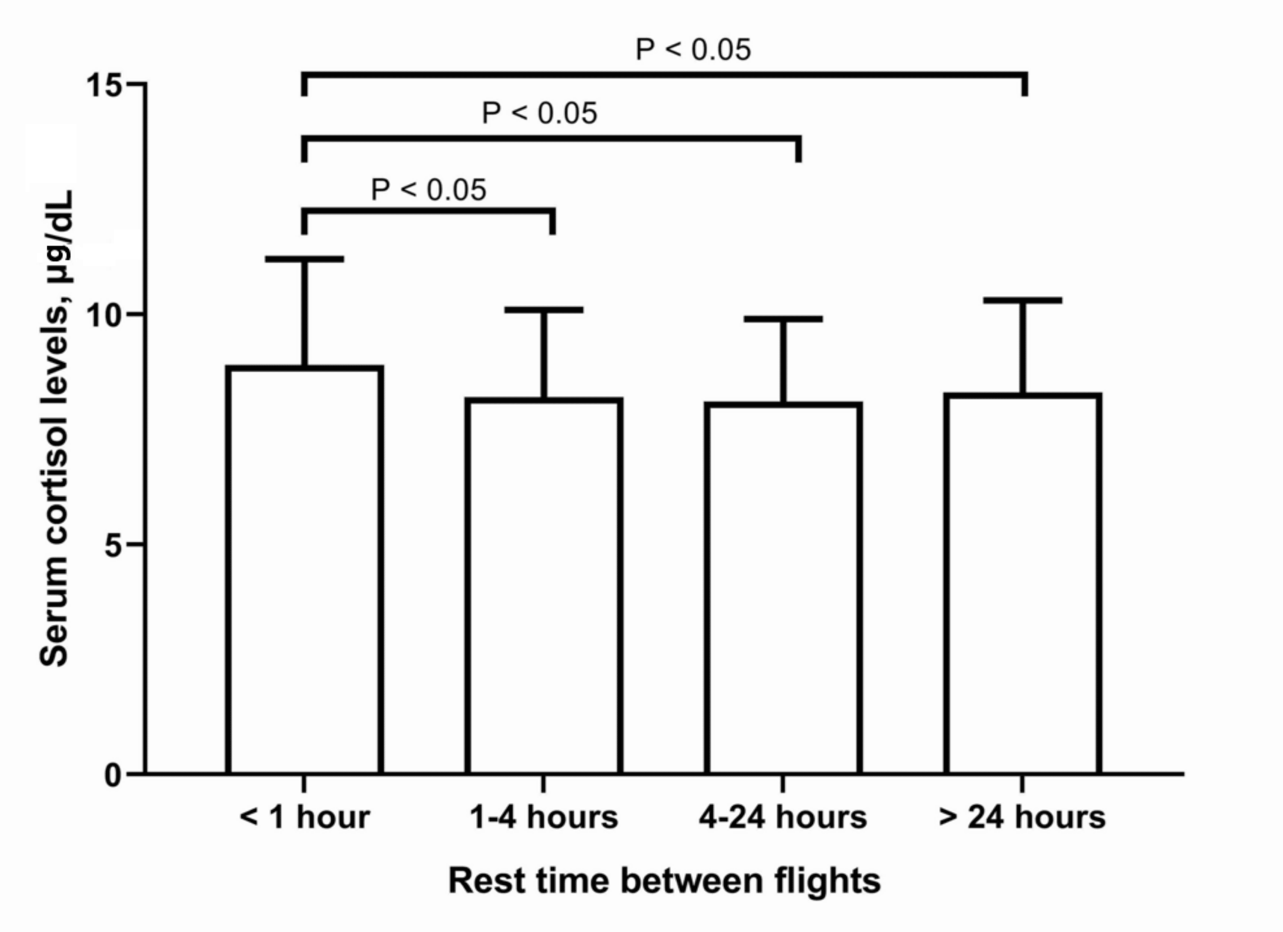
Serum cortisol levels in relation to rest time between flights in APs. Serum cortisol concentrations were measured in pilots grouped by their rest time between flights. Bars represent mean cortisol levels (μg/dL) for each rest time category, with error bars indicating SD. One-way ANOVA revealed significant intergroup differences (P<0.01). Tukey's *post hoc* tests demonstrated significant differences (P<0.05) between the <1 hour rest time group and all other groups, as indicated by the brackets. APs: airline pilots; ANOVA: analysis of variance; SD: standard deviation.

There were no significant differences in the levels of the two hormones in relation to position, years of experience, total flight hours, flight hours in the preceding year, or flight route duration. However, significant intergroup differences were observed for both cortisol (one-way ANOVA, P<0.01) and DHEAS (one-way ANOVA, P<0.001) in relation to rest time between flights. Regarding cortisol levels, the highest mean concentration was observed in pilots with the shortest rest time (<1 hour). This group differed significantly (Tukey's *post hoc* tests, P<0.05) from those with rest times of 1-4 hours, 4-24 hours, and >24 hours (Figure 1). No significant differences were observed among these three groups.

**Figure 2:**
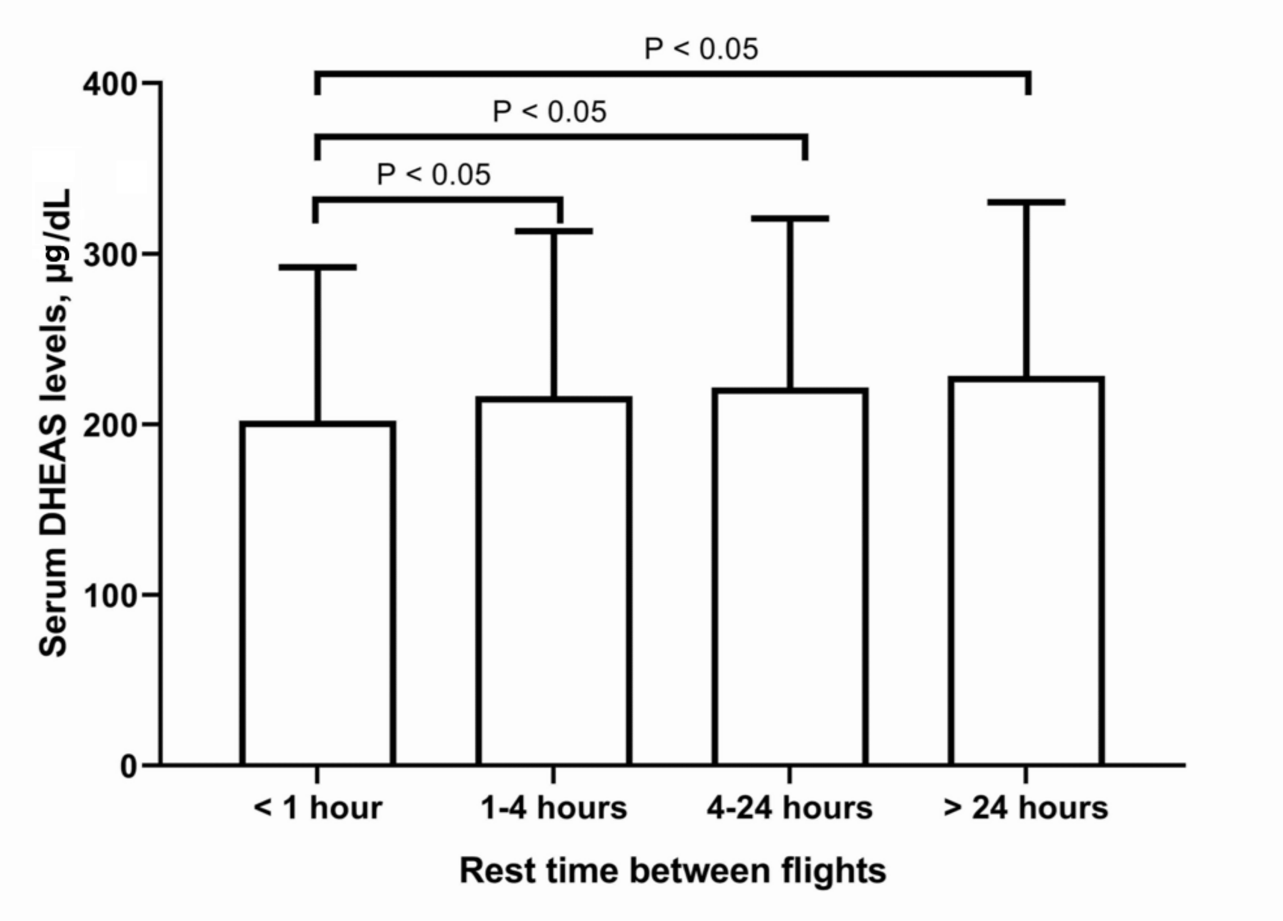
Serum DHEAS levels in relation to rest time between flights in APs. Serum DHEAS concentrations were measured in pilots grouped by their rest time between flights. Bars represent mean DHEAS levels (μg/dL) for each rest time category, with error bars indicating SD. One-way ANOVA revealed significant intergroup differences (P<0.001). A significant stepwise decrease in serum DHEAS concentrations was observed across increasing rest periods (P<0.001 for trend). Tukey's *post hoc* tests demonstrated significant differences (P<0.05) between multiple groups, as indicated by the brackets. APs: airline pilots; ANOVA: analysis of variance; DHEAS: dehydroepiandrosterone sulfate; SD: standard deviation.

Concerning DHEAS, a stepwise decrease in serum concentrations was observed across rest periods of <1 hour, 1-4 hours, 4-24 hours, and >24 hours (P<0.001 for trend; Figure 2).

Multivariable analysis

After adjustment for age, BMI, years of education, and blood pressure values in multivariable analyses, an inter-flight rest period of less than one hour remained independently associated with both serum cortisol (OR=1.09, 95% CI=1.04-1.13, P<0.01) and DHEAS (OR=0.94, 95% CI=0.92-0.97, P<0.001).

## Discussion

In this cross-sectional study involving commercial APs, we found that short inter-flight rest periods of less than one hour were independently associated with increased cortisol levels and decreased DHEAS levels, compared to longer rest durations, after controlling for potential confounding variables.

During the psychophysiological stress response, the hypothalamic-pituitary-adrenal (HPA) axis is activated by the release of corticotropin-releasing hormone from the hypothalamus, triggering a cascade of endocrine changes that culminate in the release of cortisol from the adrenal cortex [20]. In the aviation industry, cortisol has been extensively studied as a biomarker of stress in military settings and among combat pilots [21-23]. In addition, Otsuka et al. [24] reported that an increase in salivary cortisol may serve as a stress marker among student pilots. In the civilian sector, Tresguerres et al. [25] found that APs maintained their cortisol excretion patterns during both westbound and eastbound transmeridian flights. Moreover, Radowicka et al. [26] analyzed the circadian rhythm of cortisol and factors influencing its secretion among female flight attendants working within a single time zone and on long-distance flights. They concluded that while female flight attendants do not experience cortisol hypersecretion due to the nature of their work, the frequency of flying and length of work hours can affect the dysregulation of the HPA axis [26]. A notable study by Bostock and Steptoe [27] investigated the relationship between early and late flight duties, perceived stress, cortisol levels, mood, and fatigue in male short-haul pilots. The authors found that early flight duty start times were associated with higher cortisol levels upon waking and throughout the subsequent flight duty period, with significantly higher total cortisol secretion compared to later start times or rest days [27]. Our findings expand on these observations by showing that the significantly higher serum cortisol levels observed in APs with less than one hour of rest between flights suggest that extremely short turnaround times may act as an acute occupational stressor. This aligns with prior research in the field of occupational health demonstrating that insufficient recovery can impair stress regulation and lead to increased cortisol secretion [28]. Notably, chronically elevated cortisol has been linked to various adverse health outcomes, including metabolic dysregulation, immune suppression, and impaired cognitive abilities [29], which are paramount in the aviation context for adequate pilot performance.

In contrast to cortisol, serum DHEAS concentrations demonstrated a progressive decline with shorter inter-flight rest periods. DHEAS, an adrenal hormone known for its neuroprotective and immunomodulatory properties, is thought to be crucial for stress resilience [30]. Additionally, DHEAS is associated with the enhancement of cognitive function and the maintenance of brain plasticity [13]. Notably, the body's ability to produce this hormone is compromised in individuals subjected to chronic stress [14,15]. The observed decline in DHEAS with decreasing rest duration suggests that inadequate recovery time may compromise pilots' physiological resilience to stress. Furthermore, lower DHEAS concentrations have been associated with psychological distress, fatigue, and burnout [9], which could negatively impact pilot performance, especially during demanding tasks such as takeoff and landing. In addition, the observed inverse correlation between cortisol and DHEAS aligns with the concept of allostatic load [31], whereby prolonged stress exposure leads to neuroendocrine dysregulation characterized by high cortisol and low DHEAS. This hormonal imbalance may reflect a maladaptive physiological response in APs with very limited rest opportunities. Our findings underscore the importance of allowing adequate rest breaks to mitigate the neuroendocrine impact of work-related stress in pilots, which has important implications for both pilot well-being and aviation safety. Insufficient rest periods, as indicated by the observed alterations in stress hormone levels, may not only contribute to long-term health consequences among pilots but also stress-induced impairments in cognitive function and decision-making abilities, potentially jeopardizing flight safety and endangering crew members and passengers.

The main strengths of this study lie in the use of objective stress biomarkers and the examination of multiple occupational factors in a relatively large sample of commercial APs. Limitations include the cross-sectional design, which precludes conclusions about causality, and the lack of data on psychological measures or health outcomes. Larger, longitudinal studies are needed to clarify the relationships between short rest times between flights, hormonal dysregulation, and pilot health and performance over time. Another important caveat is that the study was limited to male pilots, which may limit the generalizability of the findings to female pilots. Finally, while we adjusted for several potential confounders, residual confounding by unmeasured factors cannot be ruled out. This limitation is common in studies examining hormonal interactions and their physiological impacts.

## Conclusions

This study provides novel evidence that very short inter-flight rest periods are associated with an unfavorable stress hormone profile in commercial APs, characterized by higher cortisol and lower DHEAS levels. These findings have important implications for aviation policies regarding work schedules and rest requirements. Ensuring sufficient recovery time between flights may be a key strategy to manage occupational stress, support pilot well-being, and optimize performance in this safety-critical occupation. Integrating biomarker assessments into occupational health research can provide valuable insights into the physiological impact of work-related stressors and inform evidence-based interventions to promote health and safety in the aviation industry.
